# Poisson type inequalities with respect to a cone and their applications

**DOI:** 10.1186/s13660-017-1387-y

**Published:** 2017-05-16

**Authors:** Chang Shu, Ling Chen, Robert Vargas-De-Teón

**Affiliations:** 1 Geomathematics Key Laboratory of Sichuan Province, Chengdu University of Technology, Chengdu, 610059 China; 2 Departamento de Matemáticas, Facultad de Ciencias, UNAM, México, D.F. 04510 México

**Keywords:** Poisson type inequality, harmonic function, integral representation

## Abstract

In this paper, we establish new Poisson type inequalities with respect to a cone. As applications, the integral representations of harmonic functions are also obtained.

## Introduction

Let $B(P,R)$ denote the open ball with center at *P* and radius *R* in ${\mathbf{R}}^{n}$, where ${\mathbf{R}}^{n}$ is the *n*-dimensional Euclidean space, $P\in{\mathbf{R}}^{n}$ and $R>0$. Let $B(P)$ denote the neighborhood of *P* and $S_{R}=B(O,R)$ for simplicity. The unit sphere and the upper half unit sphere in ${\mathbf{R}}^{n}$ are denoted by ${\mathbf{S}}_{1}$ and ${\mathbf{S}}_{1}^{+}$, respectively. For simplicity, a point $(1,\Theta)$ on ${\mathbf{S}}_{1}$ and the set $\{\Theta; (1,\Theta)\in\Gamma\}$ for a set Γ, $\Gamma\subset{\mathbf{S}}_{1}$, are often identified with Θ and Γ, respectively. Let $\Lambda\times\Gamma$ denote the set $\{(r,\Theta )\in{\mathbf{R}}^{n}; r\in\Lambda,(1,\Theta)\in\Gamma\}$, where $\Lambda\subset{\mathbf{R}}_{+}$ and $\Gamma\subset{\mathbf{S}}_{1}$. We denote the set ${\mathbf{R}}_{+}\times{\mathbf{S}}_{1}^{+}=\{(X,x_{n})\in{\mathbf{R}}^{n}; x_{n}>0\}$ by ${\mathbf{T}}_{n}$, which is called the half space.

We shall also write $h_{1}\approx h_{2}$ for two positive functions $h_{1}$ and $h_{2}$ if and only if there exists a positive constant *a* such that $a^{-1}h_{1}\leq h_{2}\leq ah_{1}$. We denote $\max\{u(r,\Theta),0\}$ and $\max\{-u(r,\Theta),0\}$ by $u^{+}(r,\Theta)$ and $u^{-}(r,\Theta)$, respectively.

The set ${\mathbf{R}}_{+}\times\Gamma$ in ${\mathbf{R}}^{n}$ is called a cone. We denote it by $\mathfrak{C}_{n}(\Gamma)$, where $\Gamma\subset{\mathbf{S}}_{1}$. The sets $I\times\Gamma$ and $I\times \partial{\Gamma}$ with an interval on **R** are denoted by $\mathfrak{C}_{n}(\Gamma;I)$ and $\mathfrak{S}_{n}(\Gamma;I)$, respectively. We denote $\mathfrak {C}_{n}(\Gamma)\cap S_{R}$ and $\mathfrak{S}_{n}(\Gamma; (0,+\infty))$ by $\mathfrak {S}_{n}(\Gamma; R)$ and $\mathfrak{S}_{n}(\Gamma)$, respectively.

Furthermore, we denote by *dσ* (resp. $dS_{R}$) the $(n-1)$-dimensional volume elements induced by the Euclidean metric on $\partial{\mathfrak{C}_{n}(\Gamma)}$ (resp. $S_{R}$) and by *dw* the elements of the Euclidean volume in ${\mathbf{R}}^{n}$.

It is well known (see, e.g. [1], p.41) that

1 $$ \begin{aligned} &\Delta^{*}\varphi(\Theta)+\lambda\varphi( \Theta)=0 \quad \text{in } \Gamma, \\ &\varphi(\Theta)=0 \quad \text{on } \partial{\Gamma}, \end{aligned} $$

where $\Delta^{*}$ is the Laplace-Beltrami operator. We denote the least positive eigenvalue of this boundary value problem (1) by *λ* and the normalized positive eigenfunction corresponding to *λ* by $\varphi(\Theta)$, $\int_{\Gamma}\varphi^{2}(\Theta)\,dS_{1}=1$.

We remark that the function $r^{\aleph^{\pm}}\varphi(\Theta)$ is harmonic in $\mathfrak{C}_{n}(\Gamma)$, belongs to the class $C^{2}(\mathfrak{C}_{n}(\Gamma )\backslash\{O\})$ and vanishes on $\mathfrak{S}_{n}(\Gamma)$, where

$$2\aleph^{\pm}=-n+2\pm\sqrt{(n-2)^{2}+4\lambda}. $$

For simplicity we shall write *χ* instead of $\aleph^{+}-\aleph^{-}$.

For simplicity we shall assume that the boundary of the domain Γ is twice continuously differentiable, $\varphi\in C^{2}(\overline{\Gamma})$ and $\frac{\partial\varphi}{\partial n}>0$ on *∂*Γ. Then (see [2], pp.7-8)

2 $$ \operatorname{dist}(\Theta,\partial{\Gamma})\approx\varphi(\Theta), $$

where $\Theta\in\Gamma$.

Let $\delta(P)=\operatorname{dist}(P,\partial{\mathfrak{C}_{n}(\Gamma)})$. Then

3 $$ \varphi(\Theta)\approx\delta(P), $$

for any $P=(1,\Theta)\in\Gamma$ (see [3]).

Let $u(r,\Theta)$ be a function on $\mathfrak{C}_{n}(\Gamma)$. For any given $r\in{\mathbf{R}}_{+}$, The integral

$$\int_{\Gamma}u(r,\Theta)\varphi(\Theta)\,dS_{1}, $$

is denoted by $\mathcal{N}_{u}(r)$, when it exists. The finite or infinite limit

$$\lim_{r\rightarrow\infty}r^{-\aleph^{+}}\mathcal{N}_{u}(r) $$

is denoted by $\mathscr{U}_{u}$, when it exists.

The function

$$\mathbb{P}_{\mathfrak{C}_{n}(\Gamma)}(P,Q)=\frac{\partial\mathbb {G}_{\mathfrak{C}_{n}(\Gamma)}(P,Q)}{\partial n_{Q}} $$

is called the ordinary Poisson kernel, where $\mathbb{G}_{\mathfrak{C}_{n}(\Gamma)}$ is the Green function.

The Poisson integral of *g* relative to $\mathfrak{C}_{n}(\Gamma)$ is defined by

$$\mathbb{PI}_{\mathfrak{C}_{n}(\Gamma)} [g](P)=\frac{1}{c_{n}} \int_{\mathfrak{S}_{n}(\Gamma)}\mathbb {P}_{\mathfrak{C}_{n}(\Gamma)}(P,Q)g(Q)\,d\sigma, $$

where *g* is a continuous function on $\partial{\mathfrak{C}_{n}(\Gamma)}$ and $\frac{\partial}{\partial n_{Q}}$ denotes the differentiation at *Q* along the inward normal into $\mathfrak{C}_{n}(\Gamma)$.

### Remark 1

see [4]

Let $\Gamma=S_{1}^{+}$. Then

$$\mathbb{G}_{T_{n}}(P,Q)= \textstyle\begin{cases} \log|P-Q^{\ast}|-\log|P-Q|, & n=2, \\ |P-Q|^{2-n}-|P-Q^{\ast}|^{2-n}, & n\geq3, \end{cases} $$

where $Q^{\ast}=(Y,-y_{n})$, that is, $Q^{\ast}$ is the mirror image of $Q=(Y,y_{n})$ on $\partial{T_{n}}$. Hence, for the two points $P=(X,x_{n})\in T_{n}$ and $Q=(Y,y_{n})\in\partial{T_{n}}$, we have

$$\mathbb{P}_{T_{n}}(P,Q)=\frac{\partial}{\partial n_{Q}}\mathbb{G}_{T_{n}}(P,Q)= \textstyle\begin{cases} 2|P-Q|^{-2}x_{n}, & n=2, \\ 2(n-2)|P-Q|^{-n}x_{n}, & n\geq3. \end{cases} $$

We consider functions *f* satisfying

4 $$ \int_{\mathfrak{S}_{n}(\Gamma)}\frac{|f(t,\Phi)|^{p}}{1+t^{\gamma }}\,d\sigma< \infty, $$

where $p>0$ and

$$\gamma>\frac{-\aleph^{+}-n+2}{p}+n-1. $$

Further, we denote $\mathcal{A}_{\Gamma}$ the class of all measurable functions $g(t,\Phi)$ ($Q=(t,\Phi)=(Y, y_{n})\in \mathfrak{C}_{n}(\Gamma)$) satisfying the following inequality:

5 $$ \int_{\mathfrak{C}_{n}(\Gamma)}\frac{|g(t,\Phi)|^{p}\varphi }{1+t^{\gamma+1}}\,dw< \infty $$

and the class $\mathcal{B}_{\Gamma}$ consists of all measurable functions $h(t,\Phi)$ ($(t,\Phi)=(Y, y_{n})\in\mathfrak{S}_{n}(\Gamma)$) satisfying

6 $$ \int_{\mathfrak{S}_{n}(\Gamma)}\frac{|h(t,\Phi)|^{p}}{1+t^{\gamma -1}}\frac{\partial\varphi}{\partial n}\,d\sigma< \infty. $$

We will also consider the class of all continuous functions $u(t,\Phi)$ ($(t,\Phi)\in\overline{\mathfrak{C}_{n}(\Gamma)}$) harmonic in $\mathfrak{C}_{n}(\Gamma)$ with $u^{+}(t,\Phi)\in \mathcal{A}_{\Gamma}$ ($(t,\Phi)\in\mathfrak{C}_{n}(\Gamma)$) and $u^{+}(t,\Phi)\in\mathcal{B}_{\Gamma}$ ($(t,\Phi)\in\mathfrak {S}_{n}(\Gamma)$) is denoted by $\mathcal{C}_{\Gamma}$.

### Remark 2

If we denote $\Gamma=S_{1}^{+}$ in (5) and (6), then we have

$$\int_{T_{n}}\frac{y_{n}|f(Y,y_{n})|}{1+t^{n+2}}\,dQ< \infty \quad \text{and} \quad \int_{\partial{T_{n}}}\frac{|g(Y,0)|}{1+t^{n}}\,dY< \infty. $$

### Theorem A

see [5]

*Let* *g* *be a measurable function on* $\partial{T_{n}}$ *such that*

$$\int_{\partial{T_{n}}}\frac{|g(Q)|}{1+|Q|^{n}} \,dQ< \infty. $$

*Then the harmonic function* $\mathbb{PI}_{T_{n}}[g]$ *satisfies* $\mathbb{PI}_{T_{n}}[g](P)=o(r \sec^{n-1}\theta_{1})$ *as* $r\rightarrow\infty$ *in* $T_{n}$.

## Results

We first obtain the solutions of the Dirichlet problem with continuous data on the boundary of a cone.

### Theorem 1

*Let*

$$\begin{aligned}& \aleph^{+}>\frac{\gamma -n+1}{p} \quad \textit{if } p>1, \\& \aleph^{+}\geq\gamma -n+1 \quad \textit{if } p=1, \end{aligned}$$

*and* *g* *be a continuous function on* $\partial{\mathfrak {C}_{n}(\Gamma)}$ *satisfying* (4). *Then the function* $\mathbb{PI}_{\mathfrak{C}_{n}(\Gamma)}[g](P)$ *satisfies*

$$\begin{aligned}& \mathbb{PI}_{\mathfrak{C}_{n}(\Gamma)}[g](P)\in C^{2}\bigl(\mathfrak {C}_{n}(\Gamma)\bigr)\cap C^{0}\bigl(\overline{ \mathfrak{C}_{n}(\Gamma)}\bigr), \\& \Delta\mathbb{PI}_{\mathfrak{C}_{n}(\Gamma )}[g](P)=0 \quad \textit{in } \mathfrak{C}_{n}( \Gamma), \\& \mathbb{PI}_{\mathfrak{C}_{n}(\Gamma)}[g](P)=g \quad \textit{on } \partial { \mathfrak{C}_{n}(\Gamma)}, \\& \lim_{r\rightarrow\infty, P=(r,\Theta)\in \mathfrak{C}_{n}(\Gamma)}r^{\frac{n-\gamma-1}{p}}\varphi ^{n-1}(\Theta) \mathbb{PI}_{\mathfrak{C}_{n}(\Gamma)}[g](P)=0. \end{aligned}$$

For the related results about the growth properties of $\mathbb{PI}_{T_{n}}[g](P)$ in the upper half space, we refer the reader to the paper by Zhang and Piskarev (see [6]). Corollary 1 generalizes Theorem A to the conical case.

### Corollary 1

*Let* *g* *be a continuous function on* $\partial{\mathfrak{C}_{n}(\Gamma)}$ *satisfying* (4) *with* $p=1$ *and* $\gamma=-\aleph^{-}+1$. *Then* $\mathbb{PI}_{\mathfrak{C}_{n}(\Gamma)}[g](P)$ *is a harmonic function on* $\mathfrak{C}_{n}(\Gamma)$ *and*

$$\lim_{r\rightarrow\infty, P=(r,\Theta)\in \mathfrak{C}_{n}(\Gamma)}r^{-\aleph^{+}}\varphi^{n-1}(\Theta) \mathbb{PI}_{\mathfrak{C}_{n}(\Gamma)}[g](P)=0. $$

From Theorem 1 we immediately have the following result.

### Corollary 2

*Let* *g* *be a continuous function on* $\partial{\mathfrak{C}_{n}(\Gamma)}$ *satisfying* (4) *with* $p=1$ *and* $\gamma=-\aleph^{-}+1$. *Then*

$$\mathscr{U}_{\mathbb{PI}_{\mathfrak{C}_{n}(\Gamma)}[g]}=\mathscr {U}_{\mathbb{PI}_{\mathfrak{C}_{n}(\Gamma)}[|g|]}=0. $$

It is well known that if $h\geq0$ on $T_{n}$ and $h\in\mathcal {C}_{{\mathbf{S}}_{1}^{+}}$ (see Remark 2), then [7–10] there exists a constant $c\geq0$ such that

7 $$ h(P)=\mathbb{PI}_{T_{n}}[h](P)+cx_{n} $$

for all $P=(X,x_{n})\in T_{n}$, the integral in (7) is absolutely convergent. In the half space, similar results about integral representations of analytic functions and harmonic functions were proved by Khuskivadze and Paatashvili (see [11]), Su (see [5]) and Xue (see [12]), respectively. Motivated by these results, we will prove that if $h\in\mathcal{C}_{\Gamma}$, then a similar representation to (7) also holds in $\mathfrak {C}_{n}(\Gamma)$.

### Theorem 2

*If* $h\geq0$ *on* $\mathfrak{C}_{n}(\Gamma)$ *and* $h\in\mathcal{C}_{\Gamma}$, *then* $h\in\mathcal{B}_{\Gamma}$ *and*

8 $$ h(P)=\mathbb{PI}_{\mathfrak{C}_{n}(\Gamma)}[h](P)+\mathscr {U}_{h}r^{\aleph^{+}}\varphi(\Theta) \quad \bigl(P=(r,\Theta)\in \mathfrak {C}_{n}(\Gamma)\bigr). $$

### Remark 3

Equation (8) is equivalent to (7) in the case $\Gamma={\mathbf{S}}_{1}^{+}$.

## Lemmas

The following estimates of $\mathbb{P}_{\mathfrak{C}_{n}(\Gamma)}(P,Q)$ play an important role in our discussions.

### Lemma 1

see [13], Lemma 4 and Remark

9 $$\begin{aligned}& \mathbb{P}_{\mathfrak{C}_{n}(\Gamma)}(P,Q)\leq M r^{\aleph^{-}}t^{\aleph^{+}-1} \varphi(\Theta) \end{aligned}$$

10 $$\begin{aligned}& \bigl(\textit{resp. }\mathbb{P}_{\mathfrak{C}_{n}(\Gamma)}(P,Q)\leq M r^{\aleph^{+}}t^{\aleph^{-}-1}\varphi(\Theta)\bigr), \end{aligned}$$

*where* $P \in\mathfrak{C}_{n}(\Gamma)$ *and any* $Q \in\mathfrak{S}_{n}(\Gamma)$ *such that* $0<\frac{t}{r}\leq\frac{4}{5}$ (*resp*. $0<\frac{r}{t}\leq\frac{4}{5}$);

11 $$ \mathbb{P}_{\mathfrak{C}_{n}(\Gamma)}(P,Q)\leq M\frac{\varphi(\Theta)}{t^{n-1}}+M \frac{r\varphi(\Theta)}{|P-Q|^{n}}, $$

*where* $P=(r,\Theta)\in\mathfrak{C}_{n}(\Gamma)$ *and any* $Q\in \mathfrak{S}_{n}(\Gamma; (\frac{4}{5}r,\frac{5}{4}r))$. *We have*

12 $$ \frac{\partial\mathbb{G}_{\mathfrak{C}_{n}(\Gamma ;(t_{1},t_{2}))}((t_{1},\Phi),(r,\Theta))}{\partial t}\leq M\biggl(\frac{t_{1}}{r} \biggr)^{\gamma-1}\frac{\varphi(\Phi)\varphi(\Theta)}{t_{1}^{n-1}} $$

*and*

13 $$ -M\biggl(\frac{r}{t_{2}}\biggr)^{\aleph^{+}} \frac{\varphi(\Phi)\varphi(\Theta )}{t_{2}^{n-1}}\leq\frac{\partial\mathbb{G}_{\mathfrak{C}_{n}(\Gamma ;(t_{1},t_{2}))}((t_{2},\Phi),(r,\Theta))}{\partial t}, $$

*where* $\mathbb{G}_{\mathfrak{C}_{n}(\Gamma;(t_{1},t_{2}))}$ *is the Green function of* $\mathfrak{C}_{n}(\Gamma;(t_{1},t_{2}))$ *and* $0<2t_{1}<r<\frac{1}{2}t_{2}<+\infty$.

### Lemma 2

*For* $Q'\in \partial{\mathfrak{C}_{n}(\Gamma)}$ *and any* $\epsilon>0$, *there exists a neighborhood* $B(Q')$ *of* $Q'$ *in* ${\mathbf{R}}^{n}$ *and a number* *R* ($0< R<\infty$) *such that*

14 $$ \frac{1}{c_{n}} \int_{\mathfrak{S}_{n}(\Gamma;(R,\infty))} \bigl\vert \mathbb{P}_{\mathfrak {C}_{n}(\Gamma)}(P,Q) \bigr\vert \bigl\vert g(Q) \bigr\vert \,d\sigma< \epsilon, $$

*where* $P\in\mathfrak{C}_{n}(\Gamma)\cap B(Q')$ *and* *g* *is an upper semi*-*continuous function*. *Then*

$$\limsup_{P\in\mathfrak{C}_{n}(\Gamma), P\rightarrow Q'}\mathbb{PI}_{\mathfrak{C}_{n}(\Gamma)}[g](P)\leq g \bigl(Q'\bigr). $$

### Lemma 3

see [14]

*Let* $0< r< R$ *and* $u(t,\Phi)$ *be a subharmonic function on* $\mathfrak{C}_{n}(\Gamma;(r,R))$. *Then*

$$\begin{aligned}& \int_{\mathfrak{C}_{n}(\Gamma;(r,R))} \biggl(\frac{1}{t^{\gamma -1}}-\frac{t^{\aleph^{+}}}{R^{\chi}} \biggr) \varphi\Delta u \,dw \\& \quad =\chi \int_{\mathfrak{S}_{n}(\Gamma;R)}\frac{u\varphi}{R^{1\gamma -1}} \,dS_{R} + \int_{\mathfrak{S}_{n}(\Gamma;(r,R))}u \biggl(\frac{1}{t^{\gamma -1}}-\frac{t^{\aleph^{+}}}{R^{\chi}} \biggr) \frac{\partial\varphi}{\partial n} \,d\sigma+d_{1}(r)+\frac{d_{2}(r)}{R^{\chi}}, \end{aligned}$$

*where*

$$\begin{aligned}& d_{1}(r)= \int_{\mathfrak{S}_{n}(\Gamma;r)}\frac{\aleph^{-}}{r^{1\gamma -1}}u\varphi-\frac{\varphi}{r^{\gamma-1}} \frac{\partial u}{\partial n} \,dS_{r}, \\& d_{2}(r)= \int_{\mathfrak{S}_{n}(\Gamma;r)}r^{\aleph^{+}}\varphi\frac {\partial u}{\partial n}- \frac{\aleph^{+}u\varphi}{r^{1-\aleph ^{+}}} \,dS_{r}. \end{aligned}$$

### Proof

Apply the second Green formula to the subharmonic function $u(t,\Phi)$ and

$$v(t,\Phi)= \biggl(\frac{1}{t^{\gamma-1}}-\frac{t^{\aleph ^{+}}}{R^{\chi}} \biggr)\varphi=\psi(t) \varphi $$

in the domain $\mathfrak{C}_{n}(\Gamma;(r,R))$.

Then

$$\begin{aligned} \int_{\mathfrak{C}_{n}(\Gamma;(r,R))}v \Delta u \,dw =& \int_{\mathfrak {S}_{n}(\Gamma;R)} \biggl(u\frac{\partial v}{\partial n}-v\frac {\partial u}{\partial n} \biggr)\,dS_{R}+ \int_{\mathfrak{S}_{n}(\Gamma;r)} \biggl(u\frac {\partial v}{\partial n}-v\frac{\partial u}{\partial n} \biggr)\,dS_{r} \\ &{}+ \int_{\mathfrak{S}_{n}(\Gamma;(r,R))} \biggl(u\frac{\partial v}{\partial n}-v\frac{\partial u}{\partial n} \biggr) \,d\sigma \\ =&-\psi'(R) \int_{\mathfrak{S}_{n}(\Gamma;R)}u\varphi \,dS_{R} -\psi(r) \int_{\mathfrak{S}_{n}(\Gamma;r)}\varphi\frac{\partial u}{\partial n}\,dS_{r} \\ &{}+ \psi'(r) \int_{\mathfrak{S}_{n}(\Gamma;r)}u \varphi \,dS_{r} + \int_{\mathfrak{S}_{n}(\Gamma;(r,R))}u\psi(t)\frac{\partial \varphi}{\partial n} \,d\sigma, \end{aligned}$$

which yields the desired result. □

### Lemma 4

*Let* $h(r,\Theta)$ *be a harmonic function on* $\mathfrak{C}_{n}(\Gamma)$ *vanishing continuously on* $\mathfrak {S}_{n}(\Gamma)$, *then* $h(r,\Theta)=\mathscr{U}_{h}r^{\aleph^{+}}\varphi(\Theta)$ *for* $0< r<\infty$.

### Proof

Note that $h(r,\Theta)$ is twice continuously differentiable on $\{(r,\Theta)\in{\mathbf{R}}^{n}: (1,\Theta)\in\overline{\Gamma}, 0< r<\infty\}$ (see [15], pp.101-102). By differentiating twice under the integral sign,

$$\begin{aligned} \frac{\partial^{2}\mathcal{N}_{h}(r)}{\partial r^{2}} =& \int_{\Gamma}\frac{\partial^{2}h(r,\Theta)}{\partial r^{2}}\varphi (\Theta)\,dS_{1} \\ =& -\frac{n-1}{r} \int_{\Gamma}\frac{\partial h(r,\Theta)}{\partial r}\varphi(\Theta)\,dS_{1}- \frac{1}{r^{2}} \int_{\Gamma}\bigl(\Delta^{*}h\bigr)\varphi(\Theta) \,dS_{1}. \end{aligned}$$

Hence, we obtain from the formula of Green (see, e.g. [16], p.387)

$$\int_{\Gamma}\bigl(\Delta^{*}h\bigr)\varphi(\Theta) \,dS_{1}= \int_{\Gamma}h\bigl(\Delta ^{*}\varphi(\Theta)\bigr) \,dS_{1}. $$

So

$$\frac{\partial^{2}\mathcal{N}_{h}(r)}{\partial r^{2}}+\frac {n-1}{r}\frac{\partial\mathcal{N}_{h}(r)}{\partial r}-\frac{\lambda}{r^{2}} \mathcal{N}_{h}(r)=0 $$

for any *r* ($0< r<\infty$), which gives

$$\mathcal{N}_{h}(r)=Ar^{\aleph^{+}}+Br^{\aleph^{-}}\quad (0< r< \infty), $$

where *A* and *B* are constants independent of *r*. We remark that $h(r,\Theta)$ converges uniformly to zero as $r\rightarrow0$ and hence $\lim_{r\rightarrow0}\mathcal{N}_{h}(r)=0$. Thus $A=\mathscr{U}_{h}$. Since $\mathcal{N}_{h}(r)=\mathscr {U}_{h}r^{\aleph^{+}}$, the conclusion of Lemma 4 follows immediately. □

## Proof of Theorem 1

Since the case $0< p\leq1$ can be proved similarly, we only consider the case $p>1$ here.

Let $P=(r,\Theta)\in\mathfrak{C}_{n}(\Gamma)$ be fixed. We take a number *R* such that $R>\max(1,\frac{5}{4}r)$. If $\aleph^{+}>\frac{\gamma-n+1}{p}$ and $\frac{1}{p}+\frac{1}{q}=1$, then $(\aleph^{-}+\frac{\gamma}{p}-1)q+n-1<0$. By (7), (10) and Hölder’s inequality with respect to the modified Laplace operator, we have

15 $$\begin{aligned}& \frac{1}{c_{n}} \int_{\mathfrak{S}_{n}(\Gamma;(R,\infty))} \bigl\vert \mathbb {P}_{\mathfrak{C}_{n}(\Gamma)}(P,Q) \bigr\vert \bigl\vert g(Q) \bigr\vert \,d\sigma \\& \quad \leq Mc_{n}^{-1}r^{\aleph^{+}}\varphi(\Theta) \biggl( \int_{\mathfrak{S}_{n}(\Gamma;(\frac{5}{4}r,\infty ))}t^{(\aleph^{-}+\frac{\gamma}{p}-1)q}\,d\sigma \biggr)^{\frac {1}{q}} \biggl( \int_{\mathfrak{S}_{n}(\Gamma;(R,\infty ))} \bigl\vert g(Q) \bigr\vert ^{p}t^{-\gamma} \,d\sigma \biggr)^{\frac{1}{p}} \\& \quad \leq M'r^{\frac{\gamma-n+1}{p}}\varphi(\Theta) \biggl( \int _{\mathfrak{S}_{n}(\Gamma;(R,\infty))} \bigl\vert g(Q) \bigr\vert ^{p}t^{-\gamma} \,d\sigma \biggr)^{\frac{1}{p}} \\& \quad \leq \infty, \end{aligned}$$

where

$$M'=Mc_{n}^{-1}\biggl(\frac{5}{4} \biggr)^{\aleph^{-}-1+\frac{\gamma}{p}+\frac {n-1}{q}}\biggl(\biggl(1\gamma-1-\frac{\gamma}{p}\biggr)q+1-n \biggr)^{-\frac{1}{q}}. $$

Thus $\mathbb{PI}_{\mathfrak{C}_{n}(\Gamma)}[g](P)$ is finite for any $P\in\mathfrak{C}_{n}(\Gamma)$. Since $\mathbb{P}_{\mathfrak{C}_{n}(\Gamma)}(P,Q)$ is a harmonic function of $P\in \mathfrak{C}_{n}(\Gamma)$ for any $Q\in\mathfrak{S}_{n}(\Gamma)$, $\mathbb{PI}_{\mathfrak{C}_{n}(\Gamma)}[g](P)$ is also a harmonic function of $P\in\mathfrak{C}_{n}(\Gamma)$.

Consider

$$\lim_{P\in\mathfrak{C}_{n}(\Gamma), P\rightarrow Q'}\mathbb{PI}_{\mathfrak{C}_{n}(\Gamma)}[g](P)=g \bigl(Q'\bigr) $$

for any $Q'\in \partial{\mathfrak{C}_{n}(\Gamma)}$, and apply Lemma 2 to $g(Q)$ and $-g(Q)$. Take any $Q'=(t',\Phi')\in\partial{\mathfrak{C}_{n}(\Gamma)}$ and $\epsilon>0$. Let *δ* be a positive integer. Then from (15), we can choose a number *R*, $R>\max\{1,2(t'+\delta)\}$ such that (14) holds for any $P\in\mathfrak{C}_{n}(\Gamma)\cap B(Q',\delta)$.

For *ϵ* (>0) mentioned above, there exists $R_{\epsilon}>1$ such that

$$\int_{\mathfrak{S}_{n}(\Gamma;(R_{\epsilon},\infty))}\frac {|g(Q)|^{p}}{1+t^{\gamma}}\,d\sigma< \epsilon. $$

We put $P=(r,\Theta)\in\mathfrak{C}_{n}(\Gamma)$ satisfying $r>\frac{5}{4}R_{\epsilon}$, and write

$$\mathbb{PI}_{\mathfrak{C}_{n}(\Gamma)}[g]\leq\mathbb{PI}_{1}+\mathbb {PI}_{2}+\mathbb{PI}_{3}+\mathbb{PI}_{4}+ \mathbb{PI}_{5}, $$

where

$$\begin{aligned}& \mathbb{PI}_{1}=\frac{1}{c_{n}} \int_{\mathfrak{S}_{n}(\Gamma ;(0,1])} \vert \mathbb{P}_{\mathfrak{C}_{n}(\Gamma)} \vert \vert g \vert \,d\sigma, \\& \mathbb{PI}_{2}=\frac{1}{c_{n}} \int_{\mathfrak{S}_{n}(\Gamma ;(1,R_{\epsilon}])} \vert \mathbb{P}_{\mathfrak{C}_{n}(\Gamma)} \vert \vert g \vert \,d\sigma, \\& \mathbb{PI}_{3}=\frac{1}{c_{n}} \int_{\mathfrak{S}_{n}(\Gamma;(R_{\epsilon},\frac{4}{5}r])} \vert \mathbb{P}_{\mathfrak{C}_{n}(\Gamma)} \vert \vert g \vert \,d\sigma, \\& \mathbb{PI}_{4}=\frac{1}{c_{n}} \int_{\mathfrak{S}_{n}(\Gamma;(\frac {4}{5}r,\frac{5}{4}r))} \vert \mathbb{P}_{\mathfrak{C}_{n}(\Gamma )} \vert \vert g \vert \,d\sigma, \\& \mathbb{PI}_{5}=\frac{1}{c_{n}} \int_{\mathfrak{S}_{n}(\Gamma;[\frac {5}{4}r,\infty])} \vert \mathbb{P}_{\mathfrak{C}_{n}(\Gamma)} \vert \vert g \vert \,d\sigma. \end{aligned}$$

If $\gamma>(-\aleph^{+}-n+2)p+n-1$, then $\{\aleph^{+}-1+\frac{\gamma}{p}\}q+n-1>0$. By (9) and Hölder’s inequality with respect to the modified Laplace operator we have

16 $$\begin{aligned}& \mathbb{PI}_{2}(P) \leq Mr^{\aleph^{-}}\varphi(\Theta) \int_{\mathfrak{S}_{n}(\Gamma ;(1,R_{\epsilon}])}t^{\aleph^{+}-1}\bigl|g(Q)\bigr|\,d\sigma \\& \hphantom{\mathbb{PI}_{2}(P)}\leq Mr^{\aleph^{-}}\varphi(\Theta) \biggl( \int_{\mathfrak{S}_{n}(\Gamma ;(1,R_{\epsilon}])}\bigl|g(Q)\bigr|^{p}t^{-\gamma}\,d\sigma \biggr)^{\frac {1}{p}} \biggl( \int_{\mathfrak{S}_{n}(\Gamma;(1,R_{\epsilon}])}t^{(\aleph^{+}-1+\frac{\gamma}{p})q}\,d\sigma \biggr)^{\frac {1}{q}} \\& \hphantom{\mathbb{PI}_{2}(P)}\leq M r^{\aleph^{-}}R_{\epsilon}^{\aleph^{+}+n-2+\frac{\gamma -n+1}{p}}\varphi( \Theta), \end{aligned}$$

17 $$\begin{aligned}& \mathbb{PI}_{1}(P)\leq Mr^{\aleph^{-}}\varphi( \Theta), \end{aligned}$$

18 $$\begin{aligned}& \mathbb{PI}_{3}(P)\leq M\epsilon r^{\frac{\gamma-n+1}{p}} \varphi (\Theta). \end{aligned}$$

If $\aleph^{+}>\frac{\gamma-n+1}{p}$, then $\{\aleph^{-}-1+\frac{\gamma}{p}\}q+n-1<0$. We obtain by (10) and Hölder’s inequality with respect to the modified Laplace operator

19 $$\begin{aligned} \mathbb{PI}_{5}(P) \leq& Mr^{\aleph^{+}}\varphi( \Theta) \int_{\mathfrak{S}_{n}(\Gamma;[\frac {5}{4}r,\infty))}t^{\aleph^{-}-1} \bigl\vert g(Q) \bigr\vert \,d \sigma \\ \leq& Mr^{\aleph^{+}}\varphi(\Theta) \biggl( \int_{\mathfrak{S}_{n}(\Gamma ;[\frac{5}{4}r,\infty))} \bigl\vert g(Q) \bigr\vert ^{p}t^{-\gamma} \,d\sigma \biggr)^{\frac {1}{p}} \biggl( \int_{\mathfrak{S}_{n}(\Gamma;[\frac{5}{4}r,\infty ))}t^{(\aleph^{-}-1+\frac{\gamma}{p})q}\,d\sigma \biggr)^{\frac {1}{q}} \\ \leq& M\epsilon r^{\frac{\gamma-n+1}{p}}\varphi(\Theta). \end{aligned}$$

By (11), we consider the inequality

$$\mathbb{PI}_{4}(P)\leq \mathbb{PI}_{41}(P)+ \mathbb{PI}_{42}(P), $$

where

$$\begin{aligned}& \mathbb{PI}_{41}(P)=M\varphi(\Theta) \int_{\mathfrak{S}_{n}(\Gamma ;(\frac{4}{5}r,\frac{5}{4}r))}t^{1-n} \bigl\vert g(Q) \bigr\vert \,d \sigma, \\& \mathbb{PI}_{42}(P)=Mr\varphi(\Theta) \int_{\mathfrak{S}_{n}(\Gamma ;(\frac{4}{5}r,\frac{5}{4}r))}\frac{ \vert g(Q) \vert }{ \vert P-Q \vert ^{n}}\,d\sigma. \end{aligned}$$

We first have

20 $$\begin{aligned} \mathbb{PI}_{41}(P) \leq& M\varphi(\Theta) \int_{\mathfrak{S}_{n}(\Gamma;(\frac{4}{5}r,\frac {5}{4}r))}t^{\aleph^{+}+\aleph^{-}-1} \bigl\vert g(Q) \bigr\vert \,d \sigma \\ \leq& Mr^{\aleph^{+}}\varphi(\Theta) \int_{\mathfrak{S}_{n}(\Gamma;(\frac {4}{5}r,\infty))}t^{\aleph^{-}-1} \bigl\vert g(Q) \bigr\vert \,d \sigma \\ \leq& M\epsilon r^{\frac{\gamma-n+1}{p}} \varphi(\Theta), \end{aligned}$$

which is similar to the estimate of $\mathbb{PI}_{5}(P)$.

Next, we shall estimate $\mathbb{PI}_{42}(P)$. Take a sufficiently small positive real number *b* such that $\mathfrak{S}_{n}(\Gamma;(\frac{4}{5}r,\frac{5}{4}r))\subset B(P,\frac{1}{2}r)$ for any $P=(r,\Theta)\in\Pi(b)$, where (see [10, 17, 18])

$$\Pi(b)=\Bigl\{ P=(r,\Theta)\in\mathfrak{C}_{n}(\Gamma); \inf _{z\in \partial\Gamma} \bigl\vert (1,\Theta)-(1,z) \bigr\vert < b, 0< r< \infty\Bigr\} $$

and divide $\mathfrak{C}_{n}(\Gamma)$ into two sets $\Pi(b)$ and $\mathfrak{C}_{n}(\Gamma)-\Pi(b)$.

If $P=(r,\Theta)\in\mathfrak{C}_{n}(\Gamma)-\Pi(b)$, then there exists a positive $b'$ such that $|P-Q|\geq{b}'r$ for any $Q\in \mathfrak{S}_{n}(\Gamma)$, and hence

21 $$\begin{aligned} \mathbb{PI}_{42}(P) \leq&M\varphi(\Theta) \int_{\mathfrak{S}_{n}(\Gamma;(\frac{4}{5}r,\frac {5}{4}r))}t^{1-n} \bigl\vert g(Q) \bigr\vert \,d \sigma \\ \leq& M\epsilon r^{\frac{\gamma-n+1}{p}} \varphi(\Theta). \end{aligned}$$

Put $P=(r,\Theta)\in\Pi(b)$ and set

$$H_{i}(P)=\biggl\{ Q\in\mathfrak{S}_{n}\biggl(\Gamma;\biggl( \frac{4}{5}r,\frac{5}{4}r\biggr)\biggr); 2^{i-1}\delta(P) \leq|P-Q|< 2^{i}\delta(P)\biggr\} . $$

Since $\mathfrak{S}_{n}(\Gamma)\cap\{Q\in{\mathbf{R}}^{n}: |P-Q|< \delta(P)\}=\varnothing$, we have

$$\mathbb{PI}_{42}(P)=M\sum_{i=1}^{i(P)} \int_{H_{i}(P)}r\varphi(\Theta )\frac{|g(Q)|}{|P-Q|^{n}}\,d\sigma, $$

where $i(P)$ is a positive integer satisfying $2^{i(P)-1}\delta(P)\leq\frac{r}{2}<2^{i(P)}\delta(P)$.

By (3), we have $r\varphi(\Theta)\leq M\delta(P)$ ($P=(r,\Theta)\in\mathfrak{C}_{n}(\Gamma)$). By Hölder’s inequality with respect to the modified Laplace operator (see [8]) we obtain

$$\begin{aligned}& \int_{H_{i}(P)}r\varphi(\Theta)\frac{ \vert g(Q) \vert }{ \vert P-Q \vert ^{n}}\,d\sigma \\& \quad \leq \int_{H_{i}(P)}r\varphi(\Theta)\frac{ \vert g(Q) \vert }{\{2^{i-1}\delta (P)\}^{n}}\,d\sigma \\& \quad \leq M2^{(1-i)n}\varphi^{1-n}(\Theta) \int_{H_{i}(P)}t^{1-n} \bigl\vert g(Q) \bigr\vert \,d \sigma \\& \quad \leq M\epsilon r^{\frac{\gamma-n+1}{p}}\varphi^{1-n}(\Theta) \end{aligned}$$

for $i=0,1,2,\ldots,i(P)$.

So

22 $$ \mathbb{PI}_{42}(P)\leq M\epsilon r^{\frac{\gamma-n+1}{p}} \varphi^{1-n}(\Theta). $$

Combining (16)-(22), we finally obtain

$$\mathbb{PI}_{\mathfrak{C}_{n}(\Gamma)}[g](P)=o\bigl(r^{\frac{\gamma -n+1}{p}}\varphi^{1-n}( \Theta)\bigr) $$

as $r\rightarrow\infty$, where $P=(r,\Theta)\in\mathfrak {C}_{n}(\Gamma)$. Thus we complete the proof of Theorem 1.

## Proof of Theorem 2

We apply Lemma 3 with $R>r=1$ to *h* in $\mathfrak{C}_{n}(\Gamma;(1,R))$ and have the following result (see [3, 19]):

$$\begin{aligned}& m_{+}(R)+ \int_{\mathfrak{S}_{n}(\Gamma;(1,R))}h^{+} \biggl(\frac {1}{t^{\gamma-1}}- \frac{t^{\aleph^{+}}}{R^{\chi}} \biggr) \frac{\partial\varphi}{\partial n} \,d\sigma+d_{1}+ \frac{d_{2}}{R^{\chi}} \\& \quad =m_{-}(R)+ \int_{\mathfrak{S}_{n}(\Gamma;(1,R))}h^{-} \biggl(\frac {1}{t^{\gamma-1}}- \frac{t^{\aleph^{+}}}{R^{\chi}} \biggr) \frac{\partial\varphi}{\partial n} \,d\sigma, \end{aligned}$$

where

$$\begin{aligned}& m_{\pm}(R)=\chi \int_{\mathfrak{S}_{n}(\Gamma;R)}\frac{h^{\pm }\varphi}{R^{1\gamma-1}} \,dS_{R}, \\& d_{1}= \int_{\mathfrak{S}_{n}(\Gamma;1)}\aleph^{-}h\varphi-\varphi \frac{\partial h}{\partial n}\,dS_{1}, \qquad d_{2}= \int_{\mathfrak{S}_{n}(\Gamma;1)}\varphi\frac {\partial h}{\partial n}-\aleph^{+}h \varphi \,dS_{1}. \end{aligned}$$

If $R>2$, then we have

23 $$\begin{aligned} m_{-}(R)+\frac{3}{4} \int_{\mathfrak{S}_{n}(\Gamma;(1,\frac {R}{2}))}\frac{h^{-}}{t^{\gamma-1}} \frac{\partial\varphi}{\partial n} \,d\sigma \leq&m_{-}(R)+ \int_{\mathfrak{S}_{n}(\Gamma;(1,R))}h^{-} \biggl(\frac {1}{t^{\gamma-1}}- \frac{t^{\aleph^{+}}}{R^{\chi}} \biggr) \frac{\partial\varphi}{\partial n} \,d\sigma \\ \leq& m_{+}(R)+ \int_{\mathfrak{S}_{n}(\Gamma;(1,R))}\frac{h^{+}}{t^{\gamma -1}}\frac{\partial \varphi}{\partial n} \,d \sigma+|d_{1}|+|d_{2}|. \end{aligned}$$

Since $h\in C_{\Gamma}$, we obtain by (5)

$$\int_{1}^{\infty}\frac{m_{+}(R)}{R}dR= \int_{\mathfrak{C}_{n}(\Gamma ;(1,\infty))}\frac{(h^{+})^{p}\varphi}{t^{\gamma+1}}\,d\sigma\leq 2 \int_{\mathfrak{C}_{n}(\Gamma)}\frac{(h^{+})^{p}\varphi}{1+t^{\gamma +1}}\,d\sigma< \infty, $$

which yields (see [7])

24 $$ \liminf_{R\rightarrow\infty}m_{+}(R)=0. $$

Combining (6), (22) and (23), we conclude that (see [9, 20])

$$\liminf_{R\rightarrow\infty}\frac{3}{4} \int_{\mathfrak {S}_{n}(\Gamma;(1,\frac{R}{2}))}\frac{(h^{-})^{p}}{t^{\gamma-1}} \frac{\partial\varphi}{\partial n} \,d\sigma< \infty, $$

which gives

$$\int_{\mathfrak{S}_{n}(\Gamma)}\frac{(h^{-})^{p}}{1+t^{\gamma-1}}\frac {\partial\varphi}{\partial n}\,d\sigma< \infty. $$

Notice that the condition (6) is stronger than (4), *h* also satisfies (4) by Theorem 2. Consider the harmonic function

$$h'(P)=h(P)-\mathbb{PI}_{\mathfrak{C}_{n}(\Gamma)}[h](P), $$

which vanishes continuously on $\mathfrak{S}_{n}(\Gamma)$ by Lemma 2.

Since

$$\mathscr{U}_{h'}=\mathscr{U}_{h}+\mathscr{U}_{\mathbb {PI}_{\mathfrak{C}_{n}(\Gamma)}[h]}, $$

it follows from Corollary 2 that $\mathscr{U}_{h'}=\mathscr{U}_{h}$.

Hence, by applying Lemma 4 to $h'(P)$, we obtain (8). Then Theorem 2 is proved.

## Conclusions

In this paper, we discussed the improved Poisson type inequalities with respect to a cone only using gradient information. They inherited the advantages of the Poisson type conjugate gradient methods for solving the unconstrained minimization problems, but they had broader application scope. Moreover, the integral representations of harmonic functions are also obtained.
